# Maximizing Completion of the Two-Dose COVID-19 Vaccine Series with Aid from Infographics

**DOI:** 10.3390/vaccines9111229

**Published:** 2021-10-22

**Authors:** Madison Crutcher, Paul M. Seidler

**Affiliations:** Department of Pharmacology and Pharmaceutical Sciences, University of Southern California School of Pharmacy, 1985 Zonal Ave., Los Angeles, CA 90089-9121, USA; mcrutche@usc.edu

**Keywords:** COVID-19, public health, vaccine hesitancy, vaccine confidence, vaccination, therapeutic adherence

## Abstract

Two of the three COVID-19 vaccines approved in the United States require two doses to reach full efficacy, as do others available elsewhere in the world. The complete series of multidose COVID-19 vaccines offers stronger protection against infection by SARS-CoV-2 compared to single-dose injections with the same vaccines. Achieving perfect community-level adherence is a challenge in any public health campaign, even in non-pandemic times. Vaccines requiring multiple doses combined with a surge of vaccine hesitancy and misinformation that has been witnessed by the public during the COVID-19 pandemic are exacerbating the challenge of ensuring the world’s population achieves a sufficient level of immunity against COVID-19. Here, we describe the results of our study in which we sought to determine whether completion of a two-dose COVID-19 vaccine regimen could be improved by disseminating infographics that explain what the vaccine is and why returning for the second dose is beneficial. Our results show that the proportion of COVID-19 vaccine recipients returning for a second inoculation grew after COVID-19 vaccine infographics were distributed to first-time vaccine recipients. We suggest that extending communication and outreach initiatives into the clinic positively influences the rate of follow-up visits, and that infographics are useful tools to aid and bolster the deployment of COVID-19 vaccines.

## 1. Introduction

Patient adherence to prescribed multidose pharmaceuticals is often less than perfect and non-adherence adversely effects outcomes and care [1]. Non-adherence to medications among patients with chronic illnesses is estimated to be 50% [2], as are rates of non-adherence to multidose vaccines, which are successfully completed by only 50% of individuals [3,4]. Incomplete immunization compromises individuals and entire communities by undercutting vaccine efficacy. For COVID-19, incomplete immunization is increasingly problematic in the context of the emerging variants of SARS-CoV-2, to which partially immunized individuals are more acutely vulnerable compared with individuals who completed multidose series with the same vaccines [5,6].

All but one of the COVID-19 vaccines currently in use in the world requires at least two doses to achieve the intended efficacy. The principal COVID-19 vaccines currently available include BNT162b2 (Pfizer) [7], mRNA-1273 (Moderna) [8], JNJ-78436735 (Janssen Pharmaceutical Companies) [9], AZD1222 (AstraZeneca) [10], BBIBP-CorV (Sinopharm) [11], BBV152 (Bharat Biotech) [12], CoronaVac (Sinovac) [13,14,15], and Gam-COVID-Vac (also known as Sputnik V, developed by the Gamaleya Research Institute of Epidemiology and Microbiology in Russia) [16]. Antibody responses increase following the second dose of multidose vaccines, and efficacy against variants of SARS-CoV-2 approaches levels of protection that were first reported for the original SARS-CoV-2 strain when the two-dose regimen was completed [5,6,17,18].

Non-adherence to multidose vaccine schedules stems from a variety of factors [19]. For COVID-19, the predominating factors include fear and hesitancy about the newness of mRNA vaccines [20], the sporadic unpleasant side effects associated with the immune response to the vaccine [21], and logistical constraints of scheduling and traveling to appointments [22,23]. First-time recipients of multidose COVID-19 vaccines remain prone to vaccine hesitancy even after receiving a first inoculation, and first-dose recipients may become increasingly intimidated by the prospect of robust immune responses that have been sometimes noted for recipients of the second COVID-19 vaccine inoculation. We anticipate that growing fractions of partially immunized individuals may increasingly emerge as the pandemic continues and health officials extend their reach beyond early adopters of the vaccine to holdout populations who tend to be more hesitant.

Incomplete immunization threatens to compromise the efficacy of COVID-19 vaccination. We hypothesized that adherence to two-dose COVID-19 vaccine regimens could be improved by pre-emptive action to educate patients about how the vaccine works, and why returning for a second dose is important. Infographics offer a rapid and versatile means to educate patients about vaccines and have been used in conjunction with other strategies such as short videos [24]. Consequently, we created a simple, standalone infographic about the COVID-19 vaccine and the new mRNA technology, which was offered to vaccine recipients at the time of their first inoculation. Records from one COVID-19 vaccine clinic in Los Angeles reveal greater numbers of returnees for the second dose of the two-dose COVID-19 vaccine in the weeks after the COVID-19 vaccine infographic was distributed. These results suggest that the COVID-19 infographic is effective at encouraging the return of first-time COVID-19 vaccine recipients for the second scheduled inoculation. We suggest that infographics are a means of increasing the rate of community completion of multidose COVID-19 vaccines and provide important tools for combating ongoing and future pandemics.

## 2. Materials and Methods

### 2.1. Infographic Design

**Figure 1:**
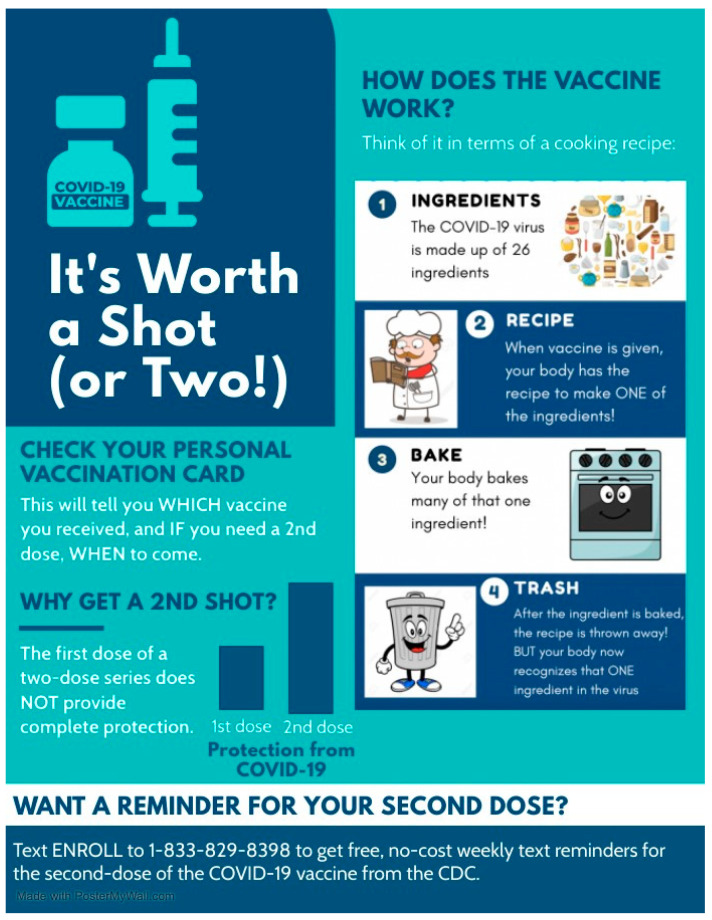
COVID-19 infographic created for distribution at the Lincoln Park COVID-19 vaccine POD in Los Angeles, CA. The infographic uses analogies and pictograms to explain in lay terms how the vaccine works.

We reasoned that heightened vaccine hesitancy due to misunderstandings about how the COVID-19 vaccine works could exacerbate the attempts of pharmacists and physicians to administer the complete series of two-dose COVID-19 vaccines to recipients. To counteract the role of vaccine hesitancy and misunderstandings in interfering with follow-up visits for the second intended dose of the two-dose COVID-19 vaccine series, the infographic in Figure 1 was created to explain, using lay terminology, how the COVID-19 vaccine works and the benefits of completing the multidose series in its entirety. In developing the content of the infographic, we sought input from community advocates and leaders in clinical pharmacy. The infographic conveys the following information to readers:

(1) The potential of two-dose COVID-19 vaccines to protect against SARS-CoV-2 is not fully realized from a single dose [7,25]. Single doses of two-dose vaccines offers variable protection ranging from 52–85% [7,25], whereas efficacies of ~95% are achieved within weeks of a second inoculation with the same vaccine [7,8]. The left side of the infographic shown in Figure 1 qualitatively illustrates these benefits to readers.
(2) COVID-19 vaccines are insufficient to cause SARS-CoV-2 infection. A study of United States residents revealed that nearly half of those surveyed thought, or were unsure about whether COVID-19 vaccines could cause infection [21]. The cartoon diagram on the right side of the infographic in Figure 1 aims to address this misinformation by explaining the COVID-19 vaccine technology with a non-scientific analogy that is comprehensible to individuals of most backgrounds and ages. The cartoon likens the 26 viral proteins [26] that are encoded by the 10 genes of the single-stranded SARS-CoV-2 genome to ingredients. Subsequent panels of the cartoon describe the COVID-19 vaccine as a recipe for a single ingredient of the 26 that would be required to make a functional virus. The COVID-19 vaccine is explained as a recipe that is received and made by recipient cells, and subsequently discarded, giving the recipient no sustained way to make the viral protein that is encoded by the vaccine. The benefit to the recipient is realized by the immune system, which learns to recognize the same viral ingredient as a threat in case of SARS-CoV-2 infection.

### 2.2. Study Design

The study was carried out at the Lincoln Park COVID-19 Vaccination Point of Dispensing (POD) in Los Angeles, CA, USA. Lincoln Park is one of the busiest PODs in Los Angeles. The Pfizer vaccine was selected as the focus of our study since it happened to be the vaccine in greatest supply at the Lincoln Park COVID-19 Vaccination POD during the duration of our study. The COVID-19 infographic in Figure 1 was made available to first-time vaccine recipients in both English and Spanish. The Spanish translation is available for download in Supplementary Figure S1. The inclusion criteria for the study included all adults visiting the Lincoln Park COVID-19 Vaccination POD as well as children aged 12 years or older, contingent upon obtaining child assent and informed consent from the guardian. Exclusion criteria for the study included patients who were receiving the Johnson and Johnson vaccine, which is a single-dose series, and patients who were visiting the site to receive the second dose of the vaccine at the time the information cards were being handed out. Participants were not actively recruited, and no compensation was provided. Participants were recruited verbally. The study design was reviewed and approved with exempt status by the University of Southern California Institutional Review Board.

### 2.3. Study Endpoints and Outcomes

**Figure 2:**
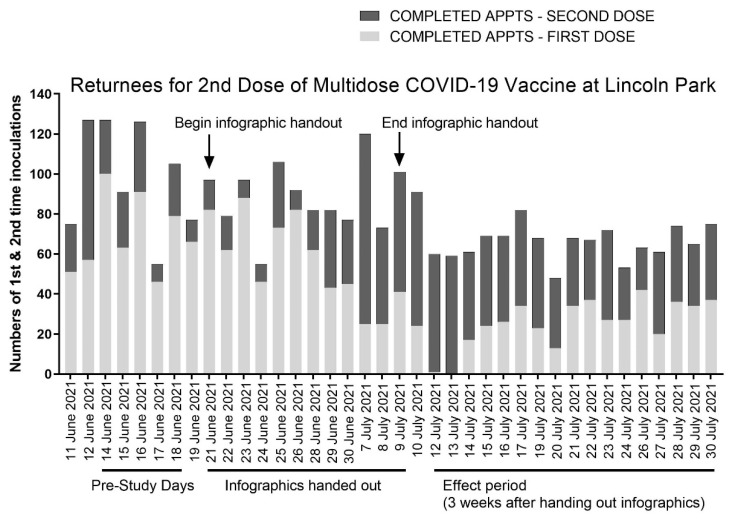
Anonymized site data from the Lincoln Park COVID-19 vaccine clinic in Los Angeles, CA, and the corresponding timeline of this study in which the COVID-19 infographic was distributed. One week prior to distributing the COVID-19 infographic (pre-study days), site records show triple the number of first-dose appointments (light gray bars) compared to second-dose appointments (dark gray bars). Three weeks after distributing infographics during the effect period, the number of second-dose appointments outnumbered first-dose appointments, on average, by 50%.

**Figure 3:**
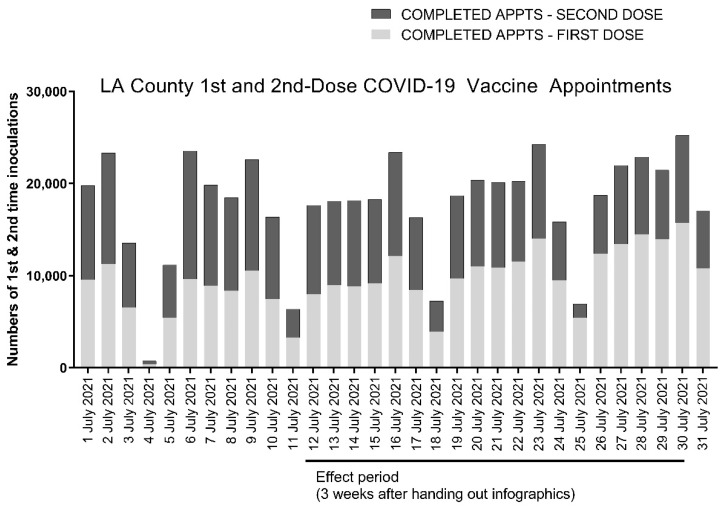
Anonymized county-wide data from the California Department of Public Health for Los Angeles County showing daily numbers of first- and second-dose recipients of the Pfizer and Moderna two-dose COVID-19 vaccines. The effect period shown corresponds to the dates of our study at the Lincoln Park COVID-19 POD from 12 to 30 July. First-dose appointments are shown with light gray bars and second-dose appointments with dark gray bars.

The study endpoint was determined based on the dates during which the COVID-19 Vaccine Infographic was handed out: 21 June through 9 July 2021. A study endpoint of 30 July was determined based on the corresponding 21 day duration from the date the last COVID-19 Vaccine Infographic was distributed, since recipients of the Pfizer vaccine are advised to return for a second inoculation 21 days after the first inoculation of the series. The number of returnees to the Lincoln Park COVID-19 Vaccination POD were determined from site records by tallying the numbers of daily completed first- and second-dose appointments for the Pfizer vaccine. Statistics were aggregated from a 7 day pre-study period to determine the typical numbers of daily first- and second-dose appointments for the Pfizer vaccine in the week preceding the initiation of our study. Statistics from the effect period were assessed from 12 to 30 July, corresponding to a window 21 days’ delayed after which the COVID-19 vaccine infographic was handed out. Raw numbers of completed first- and second-dose appointments are reported in Tables 1 and 2 and are plotted as a stacked bar plot in Figures 2 and 3. County-wide vaccine statistics were obtained from public records from the California Department of Public Health and the Los Angeles County Department of Public Health. Means of the data and variabilities, calculated as the standard deviation, are reported in the Results section.

## 3. Results

The COVID-19 infographic shown in Figure 1 was distributed between 21 June and 9 July 2021. Subsequently, we reviewed site records of 2951 visitors to the Lincoln Park COVID-19 Vaccination POD between 11 June and 30 July to determine the numbers of daily appointments serving individuals receiving first and second doses of the Pfizer COVID-19 vaccine before, during, and after the period during which COVID-19 infographics were distributed (Table 1), excluding days that fell on the weekends and over the 4 July holiday when the Lincoln Park Vaccination POD was closed.

Site records obtained from the week prior to distributing the COVID-19 vaccine infographic (14 to 18 June) reveal that, on average, 24.2% ± 5.6% of the visitors to the Lincoln Park Clinic were second-dose vaccine recipients (Figure 2, pre-study days). Appointments for first-time vaccine recipients outnumbered returnees for the second COVID-19 vaccine inoculation every day of the pre-study period by 225% to 511%.

We distributed the COVID-19 vaccine infographic between June 21st and July 9th. This was a period during which there were no vaccine supply limitations. As shown in Figure 2, three weeks after we began distributing the infographic (the duration after which individuals who received the COVID-19 infographic first became eligible to return to the clinic for their second inoculation with the Pfizer vaccine, defined as the effect period), we observed the highest fractions of returnees to the clinic. All but 1 of the 119 appointments on the first two days of the effect period, 12 and 13 July, served patients receiving a second COVID-19 vaccine inoculation.

The trend showing the increased return of patients to the clinic for their second scheduled inoculation with the COVID-19 vaccine continued for the remainder of the effect period, which spanned from 12 to 30 July, with returnees for the second vaccine dose comprising 60.9% ± 17.5% of the daily visits, on average, compared with 24.2% ± 5.6% from the pre-study period. Returnees for the second vaccine dose outnumbered first-time vaccine recipients 11 of the 15 days of the effect period. By comparison, returnees for the second vaccine dose never outnumbered first-time vaccine recipients during the week-long pre-study period at the Lincoln Park COVID-19 POD that we assessed.

We reviewed LA County records to determine the daily fraction of first- and second-dose recipients of both Pfizer and Moderna two-dose COVID-19 vaccines in surrounding communities during the window corresponding to the effect period of the study. We could not delineate recipients of the Pfizer and Moderna vaccines from county records, so these data, which are shown in Table 2 and Figure 3, include recipients of both the Pfizer and Moderna two-dose COVID-19 vaccines. The data show that slightly less than half (45.1% ± 8.1%) of individuals across Los Angeles County visiting COVID-19 vaccine sites in aggregate received a second vaccine dose during the timeframe corresponding to the effect period of our study. By comparison, the Lincoln Park COVID-19 POD served 15.8% more (60.9% ± 17.5%) second-dose recipients over the same timeframe.

**Figure 4:**
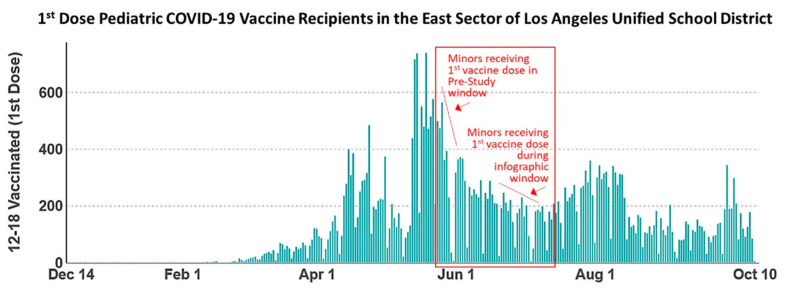
Data from the Los Angeles County Department of Public Health mined from the Community School District Data Explorer showing numbers of first-dose pediatric COVID-19 vaccine recipients in the East sector of the Los Angeles Unified School District. Pediatrics scheduled for the second COVID-19 vaccine doses during the pre-study window received a first vaccine dose between 24 and 28 May, marked on the graph accordingly. Pediatrics scheduled for the second COVID-19 vaccine dose during the effect period of the study received a first vaccine dose between 21 June and 9 July while COVID-19 vaccine infographics were being distributed, also marked on the graph accordingly. The data show a general declining trend in the numbers of pediatric COVID-19 vaccine recipients throughout the duration of this study.

Vaccine adherence in children may be higher than in adult populations and we sought to compare the numbers of pediatric vaccine recipients in the pre-study and effect period groups. Since the site records we obtained from the Lincoln Park COVID-19 POD do not distinguish vaccine recipients by age, we instead analyzed vaccine rates amongst pediatrics in the East sector of the Los Angeles Unified School District, which encompasses the Lincoln Park neighborhood. The data, shown in Figure 4, report numbers of first-dose appointments served for the pediatric population during the timeframe of our study.

Pediatric vaccine recipients scheduled for second-dose COVID-19 vaccine appointments during the pre-study window (14–18 June) received their first vaccine between 24 and 28 May. Pediatrics scheduled for the second vaccine dose during the effect period (12–30 July) of our study received their first vaccine dose between 21 June and 9 July, coinciding with distribution of the COVID-19 vaccine infographic. The data shown in Figure 4 reveal a greater number of pediatrics receiving the first dose of the COVID-19 vaccine in the timeframe between 24 and 28 May, corresponding to the pre-study group, relative to the group analyzed during the effect period who received the first COVID-19 vaccine dose between 12 and 30 July. These data show a general declining trend in the numbers of pediatric vaccine recipients throughout the study timeline, indicating that numbers of pediatrics did not remain stable throughout the study, and rates of adherence cannot be correlated with any one specific age demographic.

## 4. Discussion

COVID-19 vaccine advocates and public health officials have made tremendous strides in addressing critical issues such as maximizing accessibility to COVID-19 vaccines and dispelling misinformation that has contributed to vaccine hesitancy. Here, we aimed to extend communication and outreach beyond the focus of “getting patients in the door” for COVID-19 vaccine immunization by attempting to affect the maximal completion rate for the multidose series COVID-19 vaccines. First-time COVID-19 vaccine recipients remain subject to vaccine hesitancy and may become increasingly intimidated by the prospect of the robust immune responses that have been sometimes noted for recipients of the second COVID-19 vaccine inoculation. Thus, continued communication with vaccine recipients throughout the vaccination window, extending into and beyond the time of the first inoculation appointment, remains an important part of outreach.

Emerging studies underscore the importance of completing the two-dose vaccine series. Antibody responses are stronger and more sustained among individuals who completed the two-dose vaccine series in its entirety [5,17,18], as is protection against COVID-19 variants [5,6]. Protection from the SARS-CoV-2 delta variant, B.1.617.2, was reported to be 33% after a first inoculation with two-dose COVID-19 vaccines [6], whereas protection increases to nearly 90% following a second inoculation with the same vaccines. We suggest that continued messaging about the benefits of multidose vaccines is particularly important for bolstering community immunity in the midst of the emerging SARS-CoV-2 variants.

Regarding the limitations of this study, it is possible that additional external factors also contributed to increases in second-dose appointments seen in the effect period relative to the pre-study period that we assessed in June. For instance, we note increased numbers of returnees for the second COVID-19 vaccine beginning on 7 July, several days prior to the beginning of the effect period when we would have otherwise expected if the increase was solely predicated on whether patients received the COVID-19 infographic. We cannot rule out that the infographic and the information it conveyed were shared among visitors to the clinic, and whether that contributed to the premature increase in frequency of second-dose appointments. Another possibility is that the surge in second-dose appointments resulted from a backlog of postponed appointments in the days that preceded due to the 4 July holiday. We also note that the age of pediatric vaccine recipients steadily declined throughout our study timeframe, and the impact this may have had on adherence is uncertain.

Our analysis reveals that the Lincoln Park COVID-19 POD served 15.8% more second-dose vaccine recipients compared to the rest of Los Angeles County during the same time period. While these data offer one valuable lens through which to compare second-dose appointments at Lincoln Park with the surrounding communities, they do not account for differences that may be intrinsic to communities throughout the county with differing demographics. This limitation could have been somewhat mitigated by extending our study timeline to include an observational period at the Lincoln Park COVID-19 POD that reached several weeks beyond the effect period; however, the steadily declining rates in new vaccinations in the summer months caused consolidation of the COVID-19 Vaccine PODs in Los Angeles, resulting in closure of the Lincoln Park site on 1 August 2021. Declining vaccination enrollments can be appreciated in Figure 2. At present, daily vaccination rates in Los Angeles County remain stable at a daily rate that is about ten times lower compared to the peak vaccination rates, which occurred in March and April 2021. These statistics suggest the falloff in vaccination rates correspond with the majority of residents who were willing having now received the COVID-19 vaccine.

Our data are consistent with the interpretation that COVID-19 vaccine infographics are effective tools for increasing the rate of community completion of the multidose COVID-19 vaccine series. We found increased numbers of second-time vaccine appointments being served in the weeks that followed the distribution of our COVID-19 vaccine infographic compared to the pre-study period that we assessed. Regarding the potential impact of vaccines and the infographic on the ongoing battle against COVID-19 and its variants, breakthrough infections demonstrate a growing possible need for boosters and additional vaccines or inoculations to target emerging COVID-19 strains. Multidose vaccines remain the most effective means to suppress community infections, and infographics are a useful tool for improving community understanding and promoting completion of the full vaccine series.

## 5. Conclusions

Our data suggest that infographics are an effective way of encouraging first-time COVID-19 vaccine recipients to return for their second scheduled inoculation. Maximal efficacy, community immunity, and ultimately the end of the COVID-19 pandemic depend on the rapid and effective deployment of COVID-19 vaccines. Many communities around the world have entered a pivotal juncture in which vaccines have become available, and medical personnel are left striving to attain the highest possible rates of community immunization. Our results offer infographics as a valuable tool to aid the effective and complete deployment of multidose COVID-19 vaccines, especially in light of emerging SARS-CoV-2 variants, which attenuate vaccine efficacy for individuals with incomplete immunization.

## Figures and Tables

**Table 1:** Numbers of daily first- and second-time recipients of the Pfizer COVID-19 vaccine at the Lincoln Park Clinic in Los Angeles, CA, during the pre-study and study periods shown in Figure 2.

Date	Number of Daily Visitors	Number of 1st-Dose Recipients	Number of 2nd-Dose Recipients
11 June 2021	75	51	24
12 June 2021	127	57	70
14 June 2021	127	100	27
15 June 2021	91	63	28
16 June 2021	126	91	35
17 June 2021	55	46	9
18 June 2021	105	79	26
19 June 2021	77	66	11
21 June 2021	97	82	15
22 June 2021	79	62	17
23 June 2021	97	88	9
24 June 2021	55	46	9
25 June 2021	106	73	33
26 June 2021	92	82	10
28 June 2021	82	62	20
29 June 2021	82	43	39
30 June 2021	77	45	32
7 July 2021	120	25	95
8 July 2021	73	25	48
9 July 2021	101	41	60
10 July 2021	91	24	67
12 July 2021	60	1	59
13 July 2021	59	0	59
14 July 2021	61	17	44
15 July 2021	69	24	45
16 July 2021	69	26	43
17 July 2021	82	34	48
19 July 2021	68	23	45
20 July 2021	48	13	35
21 July 2021	68	34	34
22 July 2021	67	37	30
23 July 2021	72	27	45
24 July 2021	53	27	26
26 July 2021	63	42	21
27 July 2021	61	20	41
28 July 2021	74	36	38
29 July 2021	65	34	31
30 July 2021	75	37	38

**Table 2:** Numbers of daily first- and second-dose recipients of the Pfizer and Moderna COVID-19 vaccines in Los Angeles County in the month of July 2021. Data were obtained from the California Department of Public Health and determined by subtracting the daily summed differences of recipients of the first-dose Pfizer and Moderna vaccines from the daily totals of new fully vaccinated individuals and recipients of the Johnson and Johnson vaccine, who were considered fully vaccinated after a single dose.

Date	Number of Daily Visitors	Number of 1st-Dose Recipients	Number of 2nd-Dose Recipients
1 July 2021	19,762	9572	10,190
2 July 2021	23,318	11,238	12,080
3 July 2021	13,550	6546	7004
4 July 2021	738	383	355
5 July 2021	11,169	5445	5724
6 July 2021	23,538	9625	13,913
7 July 2021	19,823	8930	10,893
8 July 2021	18,447	8355	10,092
9 July 2021	22,603	10,516	12,087
10 July 2021	16,353	7474	8879
11 July 2021	6376	3302	3074
12 July 2021	17,646	7991	9655
13 July 2021	18,106	8983	9123
14 July 2021	18,174	8840	9334
15 July 2021	18,254	9168	9086
16 July 2021	23,386	12,153	11,233
17 July 2021	16,298	8457	7841
18 July 2021	7244	3912	3332
19 July 2021	18,689	9707	8982
20 July 2021	20,388	11,022	9366
21 July 2021	20,120	10,878	9242
22 July 2021	20,231	11,513	8718
23 July 2021	24,254	14,017	10,237
24 July 2021	15,817	9517	6300
25 July 2021	6945	5420	1525
26 July 2021	18,723	12,404	6319
27 July 2021	21,966	13,445	8521
28 July 2021	22,888	14,493	8395
29 July 2021	21,457	13,949	7508
30 July 2021	25,223	15,727	9496
31 July 2021	17,058	10,784	6274

## Data Availability

Not applicable.

## Supplementary Materials

The following are available online at https://www.mdpi.com/article/10.3390/vaccines9111229/s1, Figure S1: COVID-19 infographic from Figure 1 of the main text translated into Spanish.
