# Supplementary figures and images for: Maximizing Completion of the Two-Dose COVID-19 Vaccine Series with Aid from Infographics
Source: Vaccines (Basel). 2021 Oct 22;9(11):1229. doi: 10.3390/vaccines9111229 (PMC8618920; doi:10.3390/vaccines9111229)

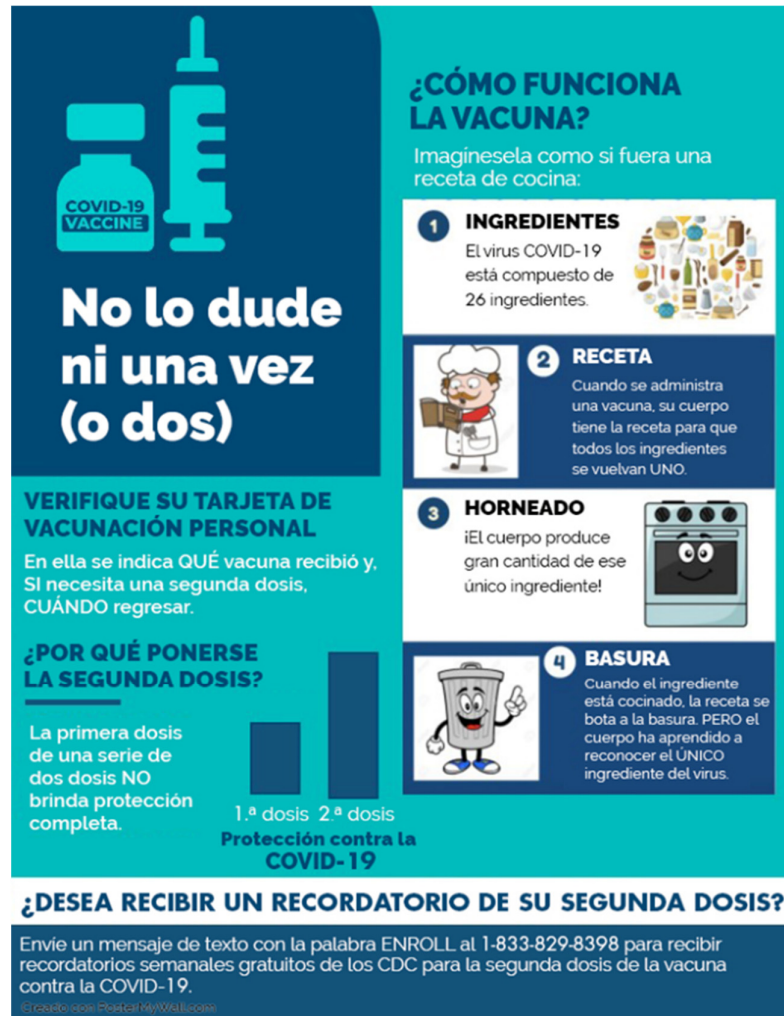

Figure S1. COVID-19 infographic from Figure 1 of the main text translated to Spanish.

Supplement: Supplementary file 1 [file vaccines-09-01229-s001.zip › vaccines-1373640-supplementary.pdf]
